# Symptomatic relief precedes improvement of myocardial blood flow in patients under spinal cord stimulation

**DOI:** 10.1186/1468-6708-6-7

**Published:** 2005-05-19

**Authors:** Holger Diedrichs, Carsten Zobel, Peter Theissen, Michael Weber, Athanassios Koulousakis, Harald Schicha, Robert HG Schwinger

**Affiliations:** 1 Department III of Internal Medicine, University of Cologne, Cologne, Germany; 2 Clinic for Stereotactic and Functional Neurosurgery, University of Cologne, Cologne, Germany; 3 Department of Nuclear Medicine, University of Cologne, Cologne, Germany

**Keywords:** Angina pectoris, spinal cord stimulation, myocardial ischemia, exercise capacity, MIBI-SPECT

## Abstract

**Background:**

Spinal cord electrical stimulation (SCS) has shown to be a treatment option for patients suffering from angina pectoris CCS III-IV although being on optimal medication and not suitable for conventional treatment strategies, e.g. CABG or PTCA. Although many studies demonstrated a clear symptomatic relief under SCS therapy, there are only a few short-term studies that investigated alterations in cardiac ischemia. Therefore doubts remain whether SCS has a direct effect on myocardial perfusion.

**Methods:**

A prospective study to investigate the short- and long-term effect of spinal cord stimulation (SCS) on myocardial ischemia in patients with refractory angina pectoris and coronary multivessel disease was designed. Myocardial ischemia was measured by MIBI-SPECT scintigraphy 3 months and 12 months after the beginning of neurostimulation. To further examine the relation between cardiac perfusion and functional status of the patients we measured exercise capacity (bicycle ergometry and 6-minute walk test), symptoms and quality of life (Seattle Angina Questionnaire [SAQ]), as well.

**Results:**

31 patients (65 ± 11 SEM years; 25 male, 6 female) were included into the study. The average consumption of short acting nitrates (SAN) decreased rapidly from 12 ± 1.6 times to 3 ± 1 times per week. The walking distance and the maximum workload increased from 143 ± 22 to 225 ± 24 meters and 68 ± 7 to 96 ± 12 watt after 3 months. Quality of life increased (SAQ) significantly after 3 month compared to baseline, as well. No further improvement was observed after one year of treament. Despite the symptomatic relief and the improvement in maximal workload computer based analysis (Emory Cardiac Toolbox) of the MIBI-SPECT studies after 3 months of treatment did not show significant alterations of myocardial ischemia compared to baseline (16 patients idem, 7 with increase and 6 with decrease of ischemia, 2 patients dropped out during initial test phase). Interestingly, in the long-term follow up after one year 16 patients (of 27 who completed the one year follow up) showed a clear decrease of myocardial ischemia and only one patient still had an increase of ischemia compared to baseline.

**Conclusion:**

Thus, spinal cord stimulation not only relieves symptoms, but reduces myocardial ischemia as well. However, since improvement in symptoms and exercise capacity starts much earlier, decreased myocardial ischemia might not be a direct effect of neurostimulation but rather be due to a better coronary collateralisation because of an enhanced physical activity of the patients.

## Introduction

In spite of continuous improvements in revascularization techniques, there remain patients ineligible for coronary artery bypass graft surgery (CABG) and coronary angioplasty. Those patients frequently suffer from severe angina, CCS (Canadian Cardiovascular Society) class III to IV; their complaints may not be managed even with optimized drug treatment. An estimated 0.5–1% of catheterized patients belongs to this category. Taking into account the above numbers of catheterizations, in Germany alone there are up to 6000 patients per year suffering from angina-related pain without an option for revascularization.

Those patients master their daily lives only by an excessive use of short-acting nitrates and avoiding physical activity. Regular use of morphine is not uncommon. A vicious circle is established: The pain limits physical activity thus inducing further decrease of physical fitness. Frequent hospitalizations, visits to the attending physician and the need for intensive care (when a myocardial infarction is suspected) cause immense costs for the health care system.

Based on the "gate control theory" [1] Wall and Sweet [2] were the first to use electric stimulation of spinal nerves or dorsal roots in treating chronic pain. This new technique called spinal cord stimulation (SCS) caused a significant decrease in ischemia-related pain and an increased perfusion of capillaries in skeletal muscle and skin through stimulating different types of neurons and fibers in the spinal cord.

In recent years the number of studies showing decreasing symptoms, improved quality of life and less signs of myocardial ischemia (mostly measured by ECG) in SCS-treated patients rose continuously [3-13]. However, the exact mode of action and the influence on myocardial perfusion are yet unknown. Very recently Jessurun and co-workers could demonstrate an improved myocardial perfusion in TENS (transcutaneous electric neurostimulation) treated patients with syndrome X after 4 weeks [14]. Besides a study with 9 patients and a 6 week follow up [15], there exist no long-term data about myocardial ischemia under SCS in patients with coronary artery disease. In our trial, we therefore investigated not only the development of symptoms, quality of life and exercise capacity but also the myocardial perfusion measured by MIBI-SPECT (single photon emission computer tomography) in a one year follow up of 31 patients with CAD and intractable angina.

## Materials and methods

### Patients

Between March 1999 and May 2003 31 patients were included in the study. Criteria for accepting a patient was a refractory angina CCS III-IV in spite of optimized medical therapy (ACE inhibitors, aspirin, β-blockers, statins, calcium channel blockers, nitrates). All patients had a coronary angiogram in the last 3 months before screening and were discussed in detail for possibility of CABG and/or PCI (percutaneus coronary intervention) by a specialized team of cardiologists and heart surgeons. Only if none of these revascularization techniques was considered as suitable was the patient included in the trial. Since relieving of pain is the main aim of SCS treatment we only included patients with angina CCS III-IV without relevant symptoms of heart failure. Hence, patients with a left ventricular ejection fraction less than 40% or a NYHA class greater than II were excluded from the study. All inclusion and exclusion criteria are listed in table 1.

**Table 1:** Inclusion and exclusion criteria

**Inclusion criteria**
Chronic coronary artery disease (CAD)
Evidence of ischemia in MIBI-SPECT examination
Coronary angiogram in the last 3 months
Angina pectoris, CCS III-IV
Optimal medical treatment (Beta-blocker, ACE-inhibitor or AT1-antagonist, Ca^2+^-antagonist, ASS, CSE-inhibitor, long-acting nitrate [LAN])
Not suitable for percutaneous coronary intervention (PCI) or aortocoronary bypass surgery (CABG) after discussion with a team of specialists (cardiologists and heart surgeons)
**Exclusion criteria**
Myocardial infarction or unstable in the last 3 months
Pacemaker with unipolar electrode
Relevant valvular heart disease
Symptomatic heart failure > NYHA II
Left ventricular ejection fraction (LVEF) < 40%

### Implantation of the electrode

The electrode was placed in the epidural space at the level of Th4-5 through a small skin incision under local anesthesia, and then it was moved up to C7-Th1 subcutaneously. Correct placement was defined by test-stimulation and interviewing the patient. Patients should experience paraesthesia in the same dermatome normally affected by angina. After final placement, the electrode was connected to an external stimulator, which allowed the patient to test the effects of SCS for 3–4 days. Afterwards the final stimulator (Medtronic Itrel III) was implanted subcutaneous in the lateral abdomen and connected to the electrode. The stimulator was controlled through an external device by the patient allowing to change the amplitude of the stimulus or to switch it on/off.

During the course of the study, examinations were scheduled after four weeks, three months (with MIBI-SPECT and SAQ), twelve month (with MIBI-SPECT and SAQ) and whenever wished by the patients.

### Exercise capacity

The bicycle ergometry protocol began at a workload of 25 Watt and increases in 25 Watt increments every 2 minutes. Exercise was terminated by the patient, when angina pectoris occurred or he was exhausted. Criteria for early termination of exercise were ischemic ST-segment depression > 2 mm; ventricular or supraventricular tachycardia; and a decrease or abnormal elevation in systolic blood pressure. In our study in none of the patients termination was necessary because of tachycardia or abnormal blood pressure.

The six-minute walk test was performed using an internal hallway with a marked distance of 50 meters. Patients were instructed to walk the distance at their own pace but to cover as much ground as possible. They were allowed to stop and rest during the test if needed.

### Nitrate intake and Seattle Angina Questionnaire (SAQ)

The SAQ is a self-administered, disease-specific measure for patients with CAD that has previously been demonstrated to be valid, reproducible, and sensitive to clinical change [16-18]. The SAQ measures five dimensions of coronary artery disease: patients' physical limitations caused by angina, the frequency of and recent changes in their symptoms (angina frequency and angina stability), their satisfaction with treatment and the degree to which they perceive their disease to affect their quality of life. Each scale is transformed to a score of 0 to 100, where higher scores indicate better function (e.g. less physical limitation, less angina, and better quality of life).

The amount of short acting nitrates (SAN) during one week was reported by the patients at every visit in the clinic. For our analysis we used average SAN consumption after 3 and 12 months.

### Myocardial scintigraphy (MIBI-SPECT)

SPECT- examinations were run on two days, one day under resting conditions, the second day after stress, using 740 MBq 99-Technetium-MIBI. Stress was induced either by physical exercise (bicycle ergometry) or by dobutamine injection (10, 20, 30, 40 μg per /kg/ min). Scanning was run one hour after the injection in the stress examinations and two hours after resting-conditions. To compare conditions before SCS implantation and during follow up stress scans were made at a comparable rate pressure product.

The camera was a three-head gamma camera with a LEHR- (low-energy-high-resolution) -collimator (prism 3000, Picker/Philips; matrix 128 × 128, 40 steps with 3° each, 20 seconds per step). For generating tomograms in short and long axis views, a filtered backprojection technique with additional semiquantitative polarmap analysis was used. SPECT examinations took place prior to the implantation of the SCS system as well as three and twelve months after.

Myocardial scintigraphy is usually used to access regional perfusion, e.g. after a heart attack or PCI. However, if the total myocardial perfusion in one patient is to be examined over a course of time, one frequently encounters the reduction of ischemia in one area combined with increased ischemia in another, e.g. improved perfusion in the anterior myocardium with reduced perfusion in the posterior myocardium. This makes it difficult to judge whether conditions have improved or actually worsened. To meet this problem, results were interpreted with the „Emory Cardiac Toolbox“ (Emory Medical Device Group). This software compares the perfusion found in the patient to a virtual normal perfusion, setting the normal nuclide distribution- that is the normal perfusion- as 1. If a patient has a score of 0.5 (50% of normal radionuclide distribution and myocardial perfusion) in the initial scan and reaches a score of 0.8 (80% of normal radionuclide distribution and myocardial perfusion) in follow-up examinations this means total perfusion has improved, even if perfusion of some areas has decreased. The score is not determined in direct comparison of two SPECT-scans, but by comparing each scan to virtual normal results. This software makes it possible to monitor the development of myocardial perfusion over a course of time.

In spite of this method, accessing the total perfusion remains more complex than judging regional perfusion (e.g. after PCI or heart attack). We therefore decided to limit our interpretation of data on the development of myocardial perfusion in the course of the study as follows: A score improved at least 0.2 was judged an improvement; a score lowered by 0.2 was regarded as a worsened state. A score of ± = 0.1 was defined as unchanged. We left out any semi-quantitative scores with standard deviation as we felt they would over-interpret the data.

### Statistical analysis

Data are presented as mean ± 95 % confidence interval. Differences among groups were compared by an unpaired t-test. Significance was assigned to a value of p < 0.05.

## Results

### Patients

The mean age was 65 years (± 11). As angina is more common in men, there were more men (n = 26) than women (n = 5) enrolled. 26 Patients suffered from a 3-vessel coronary artery disease, 5 from a 2-vessel disease. All patients had undergone bypass surgery (n = 24) and/or PCI (stent) (n = 28); five patients already underwent transmyocardial laser revascularization. Detailed patient characteristics are presented in table 2. Drug therapy had been optimized; apart from morphine intake it was not changed significantly during the trial's course (see table 3).

**Table 2:** Patient characteristics at baseline

Gender: male/female	26 / 5
Mean Age (range), years	65 (35–79)
Diagnosis	
3-vessel coronary artery disease	26 (84%)
2-vessel coronary artery disease	5 (16%)
History, number of patients and percentage	
Myocardial infarction	23 (74%)
Heart Failure >NYHA II	0 (0%)
Previous coronary intervention (PTCA/Stent)	28 (90%)
Previous CABG	24 (77%)
TMLR	5 (16%)
Cerebrovascular Disease (stroke and TIA)	5 (16%)
Symptomatic peripheral vascular disease	4 13%)
Hypertension	16 (52%)
Diabetes mellitus	7 (23%)
Hyperlipoproteinemia	23 (74%)
Adipositas (BMI >30)	8 (26%)
Nicotine abuse past/current	24 / 6 (77% / 19%)

NYHA = New York Heart Association, PTCA = percutaneous transluminal coronary angioplasty, CABG = coronary artery bypass graft, TMLR = transmyocardial laser revascularization, TIA = transitoric ischemic attack, BMI = body mass index

**Table 3:** Medication of the patients at baseline and one year follow up

	**Baseline (n = 31)**	**1 Year (n = 27)**
**Beta-blocker**	31 (100%)	27 (100%)
**ACE-Inhibior/AT_1_-Antagonist**	29 (94%)	26 (96%)
**Ca^++^-Antagonist**	26 (84%)	24 (89%)
**CSE-inhibitor**	31 (100%)	27 (100%)
**Long-acting nitrate**	31 (100%)	25 (93%)
**Morphine derivate**	12 (39%)	1 (4%)

Medication of the patients at baseline (n = 31) and one year follow up (n = 27). Before one year follow up SCS device was explanted in 3 patients (2 during test phase, one after 8 months). One patient died before last visit.

2 patients did not feel a positive effect of SCS on their symptoms in the test period (after implantation of the electrodes and using the external stimulator) and therefore dropped out during the initial phase of the study. SCS device was explanted in another patient after 8 months because of a lack of anti-anginal effect, too. Unfortunately, after explantation of the SCS device none of the 3 patients joined further follow up visits or MIBI-SPECT examinations. One patient died because of a liver cell cancer one month before one year visit. Cancer was not known at time of inclusion in the study.

### Exercise capacity

The physical fitness of patients improved as measured by six-minute walk test and bicycle ergometry already after 3 months (142.74 ± 22.13 vs. 224.83 (± 23.96) meters and 67.50 ± 7.11 vs. 96.43 ± 11.74 watt) and maintained at this level after one year (250.56 ± 25.24 meters; 98.08 ± 8.12 watt, see table 4). The 3 months follow up was finished by 29, the one year follow up by 27 patients (see above).

**Table 4:** SAN consumption per week and results of bicycle ergometry and 6-minute walk test at baseline and follow up

	**Baseline**	**3 Months**	**1 Year**
**SAN Consumption **amount/week	12.35 (± 1.6)	3.38 (± 0.96)*	2.78 (± 0.90)*
**Bicycle Ergometry **watt	67.50 (± 7.11)	96.43 (± 11.74)*	98.08 (± 8.12)*
**6-Minute Walk **meters	142.74 (± 22.13)	224.83 (± 23.96)*	250.56 (± 25.24)*

* = p < 0.05 compared to baseline. Results are listed in mean ± 95% confidence interval.

### Symptoms, nitrate intake and Seattle Angina Questionnaire (SAQ)

Apart from the three patients who decided to have the electrodes explanted as they felt no relieve in symptoms, all other patients felt a significant decrease of symptoms due to treatment with SCS (28 out of 31).

Eleven out of twelve patients treated with morphine at baseline no longer needed morphine at all; one patient was at least able to reduce the dose from 60 mg morphine sulphate (MST^®^) to 20 mg per day. Two patients no longer needed long-acting nitrates (see table 3). The use of short-acting nitrates (SAN) was lowered significantly, as well (12.35 ± 1.6 vs. 3.38 ± 0.96 and 2.78 ± 0.90 times per week, see table 4).

**Figure 1:**
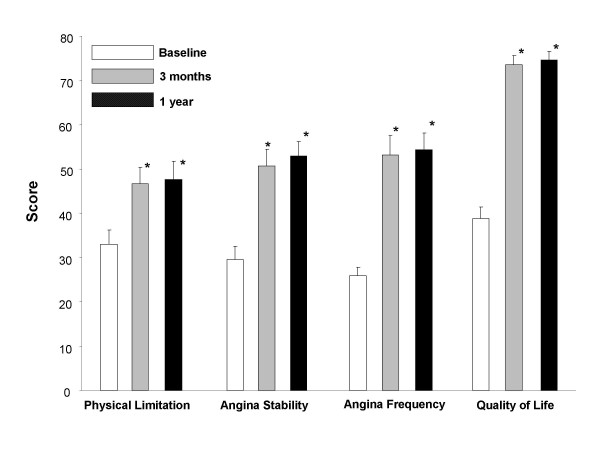
Results from the Seattle Angina Questionnaire (SAQ). * = p < 0.05 compared to baseline. Results are in mean ± 95% confidence interval (error indicator).

The Seattle Angina Questionnaire showed a significant increase in quality of Life after 3 months under SCS treatment (SAQ score 39 at baseline vs. 73). After one year the improved quality of life maintained (score 75). Scores for physical limitation 33 vs. 47 [3 months] and 48 [1 year]), angina stability (30 vs. 51 and 53) and frequency (26 vs. 53 and 54) improved significantly, as well (see figure 1).

### Myocardial perfusion (MIBI-SPECT)

**Figure 2:**
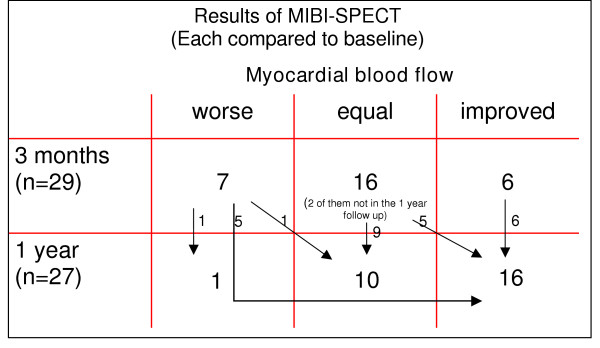
Results of the MIBI-SPECT analysis with the "Emory Cardiac Toolbox" software. Black arrows indicate the shift of the patients between the three groups (worse, equal or improved myocardial perfusion) during 3 and 12 months follow up.

**Figure 3:**
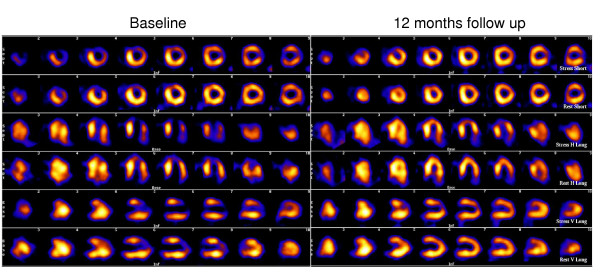
Scintigram of one patient prior to (left) and twelve months after (right) implantation. Each first line shows a stress scan, the second line the appendant rest scan. The third line shows again a stress scan etc. After one year clearly reduced hibernation resp. prolonged ischemia of the anterior LV wall (arrows vertical long axis views). Less pronounced reduction of the ischemia of the lateral LV wall (arrow heads short axis views).

As described above, data were interpreted with the „Emory Cardiac Toolbox“ software. The examinations 3 months after SCS implantation (n = 29) showed unchanged SPECT results in most patients (n = 16). Only 6 patients showed a better myocardial perfusion while in 7 ischemia worsened (figure 2). Interestingly, a shift towards an improved myocardial perfusion could be found after one year. Of 27 examined patients a worsened situation compared to baseline was found only in one patient. 16 patients showed clearly less myocardial ischemia. Results of the MIBI-SPECT analysis with the "Emory Cardiac Toolbox" software are shown in figure 2. Black arrows indicate the shift of the patients between the three groups (worse, equal or improved myocardial perfusion) during 3 and 12 months follow up. An exemplary scintigram prior to and twelve months after implantation is shown in figure 3.

### Complications

No serious complications were caused by the implantation of the neurostimulators. Neither injuries of the spinal cord nor infections occurred. The major problem in the beginning were a dislocation of the electrodes (n = 4). This could be remedied without any difficulties by surgical re-intervention. As the operating surgeons became more experienced, electrode dislocations requiring surgical intervention did not happen any longer. Small dislocations could be handled easily by re-programming the stimulation parameters.

## Discussion

This is the first clinical report with a long term follow up of myocardial perfusion measured by MIBI-SPECT in patients with multivessel coronary artery disease under spinal cord stimulation (SCS). Like in prior investigations we additionally measured symptoms, quality of life and exercise capacity of the patients, as well.

There was an early reduction of angina related symptoms under SCS treatment. Patients showed not only a significant reduction of SAN consumption (table 4) but also less use of morphine derivates already in the first weeks under neurostimulation therapy (table 3). The analysis of the self-administered Seattle Angina questionnaire (SAQ) resulted in improved scores for physical limitation, angina frequency, angina stability and quality of life after 3 months with unchanged results after one year (figure 1).

In opposite to the early functional improvement of the patients (symptoms, physical activity, quality of life), myocardial ischemia measured by MIBI-SPECT was not decreased after 3 months. 22 patients showed unchanged or even more ischemia compared to baseline before SCS implantation (figure 2). After one year – while functional parameters (exercise capacity, quality of life etc.) did not show a further change – myocardial perfusion was remarkable improved in many patients (16 of 27). Only one patient still had more cardiac ischemia than in baseline examination (figure 2). Interestingly, the score for physical limitation (SAQ) of this patient did not improve very much, as well (55 [baseline), 62 [3 months], 58 [1 year]).

Since a direct effect of SCS on myocardial perfusion should be established early after beginning of the neurostimulation, one would have expected to see alterations in the MIBI-SPECT after 3 months. The improved myocardial perfusion found after one year might therefore be due to the development of collateral vessels associated with increased physical fitness.

Two recent studies reported alterations in myocardial blood flow already after 4 or 6 weeks [22,23]. Both found a homogenization of myocardial perfusion measured by positron emission tomography (PET) in patients with multivessel disease or syndrome X. They suggested that the change in myocardial blood flow can be denoted as a steal phenomenon at the microcirculatory level eventually mediated by a direct effect of SCS on coronary vasomotion. However, they could not find an improved resting myocardial blood flow in their studies, as well. Hautvast and co-workers even found a decreased flow reserve under dipyridamole stress [22]. Thus, our study is the first to show that myocardial perfusion can improve under neurostimulation in patients with coronary multivessel disease. Since we applied a different method to measure myocardial ischemia, we cannot provide data regarding a possible homogenization of cardiac blood flow in the early phase of spinal cord stimulation. But our data suggest that the main effect of SCS treatment is anti-symptomatic and changes in myocardial ischemia are rather secondary and might be due to increased physical activity. This hypothesis is supported by data of Belardinelli and co-workers, who demonstrated that moderate exercise training improved myocardial perfusion measured by thallium scintigraphy in patients with chronic coronary artery disease [19]. If this decrease of myocardial ischemia does also improve the prognosis of the patients cannot be answered yet.

Nevertheless, the impressive improvement of angina-related symptoms, the exercise ability and last but not least the increase in quality of life of 90% (28/31) of our patients in a one year follow up supports the data of other studies which treated patients with CAD and refractory angina with spinal cord stimulation.

In a recent study, which included 32 patients with a follow up of 65 months, SCS relieved angina effectively also after long-term treatment without development of tolerance. SAQ scores still were improved after years [20]. Maintenance of increased exercise ability for years might be an ongoing stimulus for coronary collateralization.

In conclusion electrical spinal cord stimulation is a valuable therapeutic option for the treatment of refractory angina pectoris in patients not suitable for coronary revascularization procedures. Additionally, the symptomtic relief may allow more physical activity which might induce coronary collateralization resulting in an improved myocardial perfusion.

### Study limitations

Since it is difficult to conduct placebo-controlled trials with electrical neuromodulation, because there is no alternative for the paraesthesia induced by the neurostimulators, our study has -like others- no placebo group. However, measurement of myocardial perfusion with MIBI-SPECT is an objective parameter not impressionable by the patient [21,22]. As placebo effect is generally believed to decrease with time [23-26] the sustained relief of symptoms over one year in this study seem to contradict the estimation that placebo effects are the major mode of action of SCS treatment. To finally prove whether the altered myocardial perfusion is due to direct SCS effects or increased exercise ability with improved collateralization a control group without SCS but with exercise training would be necessary.
